# Supplementary material for: Studies on Silver Ions Releasing Processes and Mechanical Properties of Surface-Modified Titanium Alloy Implants
Source: Int J Mol Sci. 2018 Dec 9;19(12):3962. doi: 10.3390/ijms19123962 (PMC6321524; doi:10.3390/ijms19123962)
Supplement: Supplementary file 1 [file ijms-19-03962-s001.zip › pp11a-2000-sch_shape-13b3.rtf]

  Table 1.  Crystal data and structure refinement for pp11a-2000-sch_shape-13b3.
Identification code 	pp11a-2000-sch_shape
Empirical formula 	C15 H6 Ag5 F25 O13
Formula weight 	1408.55
Temperature 	293(2) K
Wavelength 	0.71073 Å
Crystal system 	Triclinic
Space group 	P -1
Unit cell dimensions	a = 11.3277(5) Å	a= 116.746(4)°.
	b = 13.0765(5) Å	b= 100.869(3)°.
	c = 13.7547(5) Å	g = 99.819(3)°.
Volume	1709.36(13) Å3
Z	2
Density (calculated)	2.737 Mg/m3
Absorption coefficient	3.005 mm-1
F(000)	1320
Crystal size	0.570 x 0.510 x 0.380 mm3
Theta range for data collection	2.163 to 26.371°.
Index ranges	-14<=h<=14, -16<=k<=16, -17<=l<=17
Reflections collected	18624
Independent reflections	6960 [R(int) = 0.0424]
Completeness to theta = 25.242°	99.9 % 
Absorption correction	Numerical
Max. and min. transmission	0.395 and 0.279
Refinement method	Full-matrix least-squares on F2
Data / restraints / parameters	6960 / 20 / 592
Goodness-of-fit on F2	1.043
Final R indices [I>2sigma(I)]	R1 = 0.0474, wR2 = 0.1339
R indices (all data)	R1 = 0.0627, wR2 = 0.1439
Extinction coefficient	n/a
Largest diff. peak and hole	0.973 and -0.846 e.Å-3

 Table 2.  Atomic coordinates  ( x 104) and equivalent  isotropic displacement parameters (Å2x 103)
for pp11a-2000-sch_shape-13b3.  U(eq) is defined as one third of  the trace of the orthogonalized Uij tensor.
________________________________________________________________________________ 
	x	y	z	U(eq)
________________________________________________________________________________  
Ag(1)	0	0	0	113(1)
Ag(2)	373(1)	-3373(1)	-4972(1)	55(1)
Ag(3)	95(1)	-460(1)	-4217(1)	61(1)
Ag(4)	-872(1)	-1888(1)	-2482(1)	61(1)
Ag(5)	-1931(1)	-5029(1)	-4654(1)	69(1)
Ag(6)	-4935(2)	-5423(2)	-5230(2)	129(1)
O(7)	958(4)	-570(3)	-2429(3)	52(1)
O(8)	-1476(6)	77(6)	-1853(6)	96(2)
O(9)	-3550(6)	-4834(5)	-3474(5)	98(2)
O(11)	-2186(5)	-2573(4)	-1657(4)	64(1)
O(12)	-1493(6)	-1117(5)	143(4)	86(2)
C(13)	-2173(7)	-2086(6)	-662(6)	67(2)
C(14)	-3159(10)	-2773(8)	-354(7)	95(3)
F(15)	-4240(5)	-3484(6)	-1294(5)	117(2)
F(16)	-3641(9)	-2023(6)	417(7)	166(4)
C(17)	-2681(16)	-3541(15)	18(15)	137(5)
F(18)	-3649(10)	-4078(8)	268(8)	192(4)
F(19)	-2351(11)	-4265(9)	-762(10)	179(4)
F(20)	-1817(9)	-2846(11)	964(8)	205(5)
O(21)	-3453(4)	-6741(5)	-5852(5)	78(1)
O(22)	2135(5)	-2206(4)	-3525(4)	66(1)
C(23)	3202(7)	-2341(6)	-3547(6)	66(2)
C(24)	4320(14)	-1203(12)	-2964(14)	105(9)
F(25)	5390(30)	-1290(40)	-3250(30)	320(40)
F(26)	4160(30)	-300(20)	-3170(30)	192(19)
C(27)	4871(17)	-736(17)	-1687(16)	170(20)
F(28)	3809(17)	-638(17)	-1388(13)	110(5)
F(29)	5140(30)	-1720(20)	-1740(30)	153(11)
F(30)	5891(14)	187(17)	-1243(14)	146(7)
C(64)	4256(17)	-1345(18)	-2460(16)	98(7)
F(65)	4100(30)	-610(40)	-1470(30)	330(30)
F(66)	5330(20)	-1650(20)	-2290(30)	209(17)
C(67)	4700(20)	-370(30)	-2720(20)	142(15)
F(68)	5120(30)	-960(40)	-3590(30)	203(16)
F(69)	3730(20)	40(20)	-2840(30)	191(15)
F(70)	5651(19)	515(16)	-1823(16)	175(9)
O(31)	974(4)	-5017(4)	-3485(4)	66(1)
O(32)	-235(4)	-3806(4)	-3131(4)	63(1)
C(33)	696(6)	-4122(5)	-2897(5)	53(1)
C(34)	1666(8)	-3267(7)	-1701(7)	78(2)
F(35)	2915(5)	-3252(6)	-1777(5)	114(2)
F(36)	1618(5)	-2142(4)	-1316(4)	99(2)
C(37)	1633(15)	-3611(11)	-858(9)	128(5)
F(38)	1762(10)	-4654(7)	-1143(6)	162(3)
F(39)	2502(7)	-2843(7)	134(5)	149(3)
F(40)	449(7)	-3580(10)	-749(6)	169(4)
O(41)	811(5)	-1710(4)	-5524(4)	66(1)
O(42)	-655(5)	1014(4)	-3262(4)	62(1)
C(43)	-903(6)	1707(5)	-3601(5)	54(1)
C(44)	-1434(8)	2672(7)	-2819(7)	71(2)
F(45)	-1150(8)	3676(5)	-2915(7)	143(3)
F(46)	-927(6)	3041(6)	-1719(4)	116(2)
C(47)	-2806(9)	2258(11)	-3098(9)	96(3)
F(48)	-3237(7)	3119(7)	-2426(6)	132(2)
F(49)	-3104(7)	1362(7)	-2951(10)	178(4)
F(50)	-3322(8)	1917(12)	-4136(7)	220(6)
O(51)	-1376(4)	-2401(4)	-4535(4)	56(1)
O(52)	-3088(5)	-3850(6)	-5082(7)	101(2)
C(53)	-2497(6)	-2970(6)	-5057(6)	55(1)
C(54)	-3278(7)	-2451(8)	-5651(8)	85(2)
F(55)	-2856(7)	-1225(5)	-5099(7)	142(3)
F(56)	-4503(5)	-2700(6)	-5665(5)	107(2)
C(57)	-3279(12)	-2844(16)	-6812(12)	134(5)
F(58)	-3814(11)	-4034(9)	-7372(8)	198(5)
F(59)	-2150(6)	-2731(11)	-6888(7)	177(4)
F(60)	-3934(7)	-2345(9)	-7286(7)	162(3)
________________________________________________________________________________ 
 Table 3.   Bond lengths [Å] and angles [°] for  pp11a-2000-sch_shape-13b3.
_____________________________________________________ 
Ag(1)-O(12) 	2.138(5)
Ag(1)-O(12)#1 	2.138(5)
Ag(1)-Ag(4)#1 	3.0058(5)
Ag(1)-Ag(4) 	3.0058(5)
Ag(2)-O(31)#2 	2.219(5)
Ag(2)-O(22) 	2.237(5)
Ag(2)-O(51) 	2.553(4)
Ag(2)-Ag(5)#2 	2.8950(7)
Ag(2)-Ag(5) 	3.3236(8)
Ag(3)-O(42) 	2.205(4)
Ag(3)-O(41) 	2.218(4)
Ag(3)-O(7) 	2.547(4)
Ag(3)-O(51) 	2.588(4)
Ag(3)-Ag(3)#3 	2.8932(9)
Ag(4)-O(11) 	2.324(4)
Ag(4)-O(7) 	2.426(4)
Ag(4)-O(51) 	2.511(4)
Ag(4)-O(32) 	2.540(5)
Ag(4)-O(8) 	2.577(6)
Ag(5)-O(21) 	2.234(5)
Ag(5)-O(32) 	2.249(4)
Ag(5)-O(52) 	2.379(5)
Ag(5)-Ag(2)#2 	2.8951(7)
Ag(5)-Ag(6) 	3.239(2)
Ag(6)-O(9) 	2.318(7)
Ag(6)-O(9)#4 	2.412(8)
Ag(6)-O(52)#4 	2.472(6)
Ag(6)-O(52) 	2.579(6)
Ag(6)-O(21) 	2.593(5)
O(11)-C(13) 	1.218(8)
O(12)-C(13) 	1.228(8)
C(13)-C(14) 	1.554(10)
C(14)-F(16) 	1.360(11)
C(14)-F(15) 	1.403(11)
C(14)-C(17) 	1.451(18)
C(17)-F(19) 	1.243(17)
C(17)-F(20) 	1.276(17)
C(17)-F(18) 	1.382(15)
O(21)-C(23)#2 	1.232(9)
O(22)-C(23) 	1.256(8)
C(23)-O(21)#2 	1.232(9)
C(23)-C(24) 	1.537(5)
C(23)-C(64) 	1.549(15)
C(24)-F(25) 	1.357(10)
C(24)-F(26) 	1.357(10)
C(24)-C(27) 	1.529(17)
C(27)-F(30) 	1.326(16)
C(27)-F(28) 	1.350(17)
C(27)-F(29) 	1.343(19)
C(64)-F(66) 	1.359(18)
C(64)-F(65) 	1.332(19)
C(64)-C(67) 	1.506(19)
C(67)-F(70) 	1.34(3)
C(67)-F(69) 	1.322(19)
C(67)-F(68) 	1.332(18)
O(31)-C(33) 	1.221(8)
O(31)-Ag(2)#2 	2.219(5)
O(32)-C(33) 	1.236(8)
C(33)-C(34) 	1.551(9)
C(34)-F(36) 	1.336(9)
C(34)-C(37) 	1.425(14)
C(34)-F(35) 	1.435(10)
C(37)-F(38) 	1.281(14)
C(37)-F(39) 	1.327(12)
C(37)-F(40) 	1.383(17)
O(41)-C(43)#3 	1.230(7)
O(42)-C(43) 	1.239(7)
C(43)-O(41)#3 	1.230(7)
C(43)-C(44) 	1.547(10)
C(44)-F(46) 	1.331(9)
C(44)-F(45) 	1.368(9)
C(44)-C(47) 	1.469(11)
C(47)-F(50) 	1.266(12)
C(47)-F(49) 	1.278(13)
C(47)-F(48) 	1.335(12)
O(51)-C(53) 	1.239(7)
O(52)-C(53) 	1.211(8)
C(53)-C(54) 	1.529(10)
C(54)-F(55) 	1.372(11)
C(54)-F(56) 	1.366(10)
C(54)-C(57) 	1.441(16)
C(57)-F(59) 	1.291(14)
C(57)-F(60) 	1.329(14)
C(57)-F(58) 	1.343(18)

O(12)-Ag(1)-O(12)#1	180.0
O(12)-Ag(1)-Ag(4)#1	95.09(12)
O(12)#1-Ag(1)-Ag(4)#1	84.91(12)
O(12)-Ag(1)-Ag(4)	84.91(12)
O(12)#1-Ag(1)-Ag(4)	95.09(12)
Ag(4)#1-Ag(1)-Ag(4)	180.0
O(31)#2-Ag(2)-O(22)	159.63(18)
O(31)#2-Ag(2)-O(51)	91.49(16)
O(22)-Ag(2)-O(51)	108.03(16)
O(31)#2-Ag(2)-Ag(5)#2	81.38(12)
O(22)-Ag(2)-Ag(5)#2	78.26(13)
O(51)-Ag(2)-Ag(5)#2	163.39(10)
O(31)#2-Ag(2)-Ag(5)	61.33(14)
O(22)-Ag(2)-Ag(5)	120.51(13)
O(51)-Ag(2)-Ag(5)	67.06(10)
Ag(5)#2-Ag(2)-Ag(5)	96.42(2)
O(42)-Ag(3)-O(41)	162.69(16)
O(42)-Ag(3)-O(7)	91.16(14)
O(41)-Ag(3)-O(7)	104.90(15)
O(42)-Ag(3)-O(51)	106.76(16)
O(41)-Ag(3)-O(51)	84.01(16)
O(7)-Ag(3)-O(51)	75.23(13)
O(42)-Ag(3)-Ag(3)#3	82.99(11)
O(41)-Ag(3)-Ag(3)#3	79.70(11)
O(7)-Ag(3)-Ag(3)#3	158.57(10)
O(51)-Ag(3)-Ag(3)#3	126.20(10)
O(11)-Ag(4)-O(7)	153.99(15)
O(11)-Ag(4)-O(51)	126.55(15)
O(7)-Ag(4)-O(51)	78.78(14)
O(11)-Ag(4)-O(32)	88.59(16)
O(7)-Ag(4)-O(32)	100.33(14)
O(51)-Ag(4)-O(32)	85.85(15)
O(11)-Ag(4)-O(8)	93.10(17)
O(7)-Ag(4)-O(8)	78.17(17)
O(51)-Ag(4)-O(8)	93.07(17)
O(32)-Ag(4)-O(8)	178.31(16)
O(11)-Ag(4)-Ag(1)	74.81(10)
O(7)-Ag(4)-Ag(1)	79.64(9)
O(51)-Ag(4)-Ag(1)	148.61(10)
O(32)-Ag(4)-Ag(1)	120.40(11)
O(8)-Ag(4)-Ag(1)	60.22(15)
O(21)-Ag(5)-O(32)	155.4(2)
O(21)-Ag(5)-O(52)	93.4(2)
O(32)-Ag(5)-O(52)	108.9(2)
O(21)-Ag(5)-Ag(2)#2	82.41(13)
O(32)-Ag(5)-Ag(2)#2	81.11(12)
O(52)-Ag(5)-Ag(2)#2	156.0(2)
O(21)-Ag(5)-Ag(6)	52.75(13)
O(32)-Ag(5)-Ag(6)	136.00(13)
O(52)-Ag(5)-Ag(6)	51.94(14)
Ag(2)#2-Ag(5)-Ag(6)	133.99(5)
O(21)-Ag(5)-Ag(2)	134.02(15)
O(32)-Ag(5)-Ag(2)	61.71(13)
O(52)-Ag(5)-Ag(2)	82.47(13)
Ag(2)#2-Ag(5)-Ag(2)	83.578(19)
Ag(6)-Ag(5)-Ag(2)	133.31(6)
O(9)-Ag(6)-O(9)#4	154.41(13)
O(9)-Ag(6)-O(52)#4	98.8(3)
O(9)#4-Ag(6)-O(52)#4	78.7(2)
O(9)-Ag(6)-O(52)	78.3(2)
O(9)#4-Ag(6)-O(52)	93.6(2)
O(52)#4-Ag(6)-O(52)	156.09(11)
O(9)-Ag(6)-O(21)	79.1(2)
O(9)#4-Ag(6)-O(21)	124.0(2)
O(52)#4-Ag(6)-O(21)	122.1(2)
O(52)-Ag(6)-O(21)	81.03(19)
O(9)-Ag(6)-Ag(5)	53.70(16)
O(9)#4-Ag(6)-Ag(5)	134.01(17)
O(52)#4-Ag(6)-Ag(5)	147.11(18)
O(52)-Ag(6)-Ag(5)	46.58(12)
O(21)-Ag(6)-Ag(5)	43.30(11)
Ag(4)-O(7)-Ag(3)	100.80(14)
C(13)-O(11)-Ag(4)	127.7(4)
C(13)-O(12)-Ag(1)	123.2(4)
O(11)-C(13)-O(12)	129.3(6)
O(11)-C(13)-C(14)	116.1(6)
O(12)-C(13)-C(14)	114.6(6)
F(16)-C(14)-F(15)	101.2(9)
F(16)-C(14)-C(17)	111.5(10)
F(15)-C(14)-C(17)	108.2(9)
F(16)-C(14)-C(13)	111.9(7)
F(15)-C(14)-C(13)	110.7(6)
C(17)-C(14)-C(13)	112.7(10)
F(19)-C(17)-F(20)	115.8(18)
F(19)-C(17)-F(18)	113.5(13)
F(20)-C(17)-F(18)	106.5(13)
F(19)-C(17)-C(14)	108.1(12)
F(20)-C(17)-C(14)	106.2(13)
F(18)-C(17)-C(14)	106.0(13)
C(23)#2-O(21)-Ag(5)	120.8(4)
C(23)#2-O(21)-Ag(6)	154.6(5)
Ag(5)-O(21)-Ag(6)	83.95(17)
C(23)-O(22)-Ag(2)	123.9(4)
O(21)#2-C(23)-O(22)	127.7(6)
O(21)#2-C(23)-C(24)	113.7(9)
O(22)-C(23)-C(24)	117.2(10)
O(21)#2-C(23)-C(64)	117.5(10)
O(22)-C(23)-C(64)	112.3(9)
F(25)-C(24)-F(26)	97(3)
F(25)-C(24)-C(27)	98(2)
F(26)-C(24)-C(27)	111.3(17)
F(25)-C(24)-C(23)	118(2)
F(26)-C(24)-C(23)	117.1(13)
C(27)-C(24)-C(23)	112.7(11)
F(30)-C(27)-F(28)	122.9(19)
F(30)-C(27)-F(29)	112(2)
F(28)-C(27)-F(29)	110(2)
F(30)-C(27)-C(24)	110.3(16)
F(28)-C(27)-C(24)	98.3(15)
F(29)-C(27)-C(24)	99.0(17)
F(66)-C(64)-F(65)	111(3)
F(66)-C(64)-C(67)	100(2)
F(65)-C(64)-C(67)	95(3)
F(66)-C(64)-C(23)	113.2(15)
F(65)-C(64)-C(23)	126.3(19)
C(67)-C(64)-C(23)	106.2(17)
F(70)-C(67)-F(69)	109(2)
F(70)-C(67)-F(68)	111(2)
F(69)-C(67)-F(68)	122(3)
F(70)-C(67)-C(64)	109.6(19)
F(69)-C(67)-C(64)	105(2)
F(68)-C(67)-C(64)	100(3)
C(33)-O(31)-Ag(2)#2	124.1(4)
C(33)-O(32)-Ag(5)	123.0(4)
C(33)-O(32)-Ag(4)	138.3(4)
Ag(5)-O(32)-Ag(4)	98.30(17)
O(31)-C(33)-O(32)	128.9(6)
O(31)-C(33)-C(34)	114.7(6)
O(32)-C(33)-C(34)	116.4(6)
F(36)-C(34)-C(37)	111.1(8)
F(36)-C(34)-F(35)	106.2(7)
C(37)-C(34)-F(35)	101.7(9)
F(36)-C(34)-C(33)	111.0(6)
C(37)-C(34)-C(33)	116.2(7)
F(35)-C(34)-C(33)	109.7(6)
F(38)-C(37)-F(39)	107.4(10)
F(38)-C(37)-F(40)	110.7(12)
F(39)-C(37)-F(40)	110.0(12)
F(38)-C(37)-C(34)	114.4(11)
F(39)-C(37)-C(34)	112.4(10)
F(40)-C(37)-C(34)	101.9(10)
C(43)#3-O(41)-Ag(3)	126.1(4)
C(43)-O(42)-Ag(3)	122.3(4)
O(41)#3-C(43)-O(42)	128.9(6)
O(41)#3-C(43)-C(44)	116.6(5)
O(42)-C(43)-C(44)	114.5(5)
F(46)-C(44)-F(45)	105.3(7)
F(46)-C(44)-C(47)	108.4(7)
F(45)-C(44)-C(47)	107.5(8)
F(46)-C(44)-C(43)	112.2(6)
F(45)-C(44)-C(43)	110.6(5)
C(47)-C(44)-C(43)	112.5(7)
F(50)-C(47)-F(49)	107.7(13)
F(50)-C(47)-F(48)	110.1(8)
F(49)-C(47)-F(48)	108.4(9)
F(50)-C(47)-C(44)	110.2(9)
F(49)-C(47)-C(44)	109.4(8)
F(48)-C(47)-C(44)	111.0(9)
C(53)-O(51)-Ag(4)	111.1(4)
C(53)-O(51)-Ag(2)	121.5(4)
Ag(4)-O(51)-Ag(2)	96.40(14)
C(53)-O(51)-Ag(3)	134.8(4)
Ag(4)-O(51)-Ag(3)	97.46(15)
Ag(2)-O(51)-Ag(3)	87.61(13)
C(53)-O(52)-Ag(5)	117.4(4)
C(53)-O(52)-Ag(6)	161.1(5)
Ag(5)-O(52)-Ag(6)	81.48(16)
O(52)-C(53)-O(51)	127.5(6)
O(52)-C(53)-C(54)	114.8(6)
O(51)-C(53)-C(54)	117.4(6)
F(55)-C(54)-F(56)	104.4(8)
F(55)-C(54)-C(57)	105.3(10)
F(56)-C(54)-C(57)	107.1(8)
F(55)-C(54)-C(53)	112.1(7)
F(56)-C(54)-C(53)	112.6(7)
C(57)-C(54)-C(53)	114.5(10)
F(59)-C(57)-F(60)	113.8(11)
F(59)-C(57)-F(58)	103.9(15)
F(60)-C(57)-F(58)	109.9(11)
F(59)-C(57)-C(54)	111.0(10)
F(60)-C(57)-C(54)	112.7(14)
F(58)-C(57)-C(54)	104.9(10)
_____________________________________________________________ 
Symmetry transformations used to generate equivalent atoms: 
#1 -x,-y,-z    #2 -x,-y-1,-z-1    #3 -x,-y,-z-1      
#4 -x-1,-y-1,-z-1      

 Table 4.   Anisotropic displacement parameters  (Å2x 103) for pp11a-2000-sch_shape-13b3.  The anisotropic
displacement factor exponent takes the form:  -2p2[ h2 a*2U11 + ...  + 2 h k a* b* U12 ]
______________________________________________________________________________ 
	U11	U22 	U33	U23	U13	U12
______________________________________________________________________________ 
Ag(1)	145(1) 	72(1)	54(1) 	0(1)	40(1) 	-48(1)
Ag(2)	59(1) 	48(1)	52(1) 	21(1)	12(1) 	18(1)
Ag(3)	86(1) 	53(1)	61(1) 	34(1)	35(1) 	30(1)
Ag(4)	78(1) 	49(1)	52(1) 	23(1)	25(1) 	10(1)
Ag(5)	61(1) 	50(1)	81(1) 	23(1)	12(1) 	19(1)
Ag(6)	48(1) 	168(3)	152(2) 	69(2)	28(1) 	21(1)
O(7)	61(2) 	42(2)	45(2) 	18(2)	15(2) 	13(2)
O(8)	116(5) 	111(5)	124(5) 	86(4)	70(4) 	60(4)
O(9)	121(5) 	68(3)	76(4) 	19(3)	42(3) 	-6(3)
O(11)	79(3) 	47(2)	49(2) 	14(2)	24(2) 	-2(2)
O(12)	113(4) 	61(3)	47(3) 	12(2)	28(3) 	-19(3)
C(13)	84(5) 	46(3)	51(4) 	13(3)	29(3) 	-7(3)
C(14)	130(8) 	62(5)	58(4) 	12(4)	40(5) 	-17(5)
F(15)	91(3) 	134(5)	109(4) 	65(4)	22(3) 	-13(3)
F(16)	217(8) 	107(5)	181(7) 	48(5)	153(7) 	29(5)
C(17)	155(12) 	122(11)	141(13) 	86(10)	39(11) 	3(10)
F(18)	258(10) 	151(7)	182(8) 	111(6)	94(7) 	-18(7)
F(19)	261(12) 	133(7)	231(11) 	125(8)	140(10) 	90(8)
F(20)	172(7) 	256(11)	164(8) 	157(9)	-34(7) 	-49(8)
O(21)	55(3) 	65(3)	84(4) 	16(3)	15(2) 	16(2)
O(22)	73(3) 	54(3)	49(2) 	10(2)	10(2) 	17(2)
C(23)	58(4) 	51(4)	58(4) 	10(3)	8(3) 	1(3)
C(24)	91(14) 	86(15)	55(12) 	-11(11)	23(11) 	-35(11)
F(25)	230(30) 	350(60)	100(20) 	-30(30)	90(20) 	-180(40)
F(26)	170(20) 	111(16)	230(30) 	130(20)	-80(20) 	-77(15)
C(27)	90(18) 	160(30)	86(17) 	-55(19)	35(15) 	-59(19)
F(28)	90(11) 	123(11)	83(8) 	38(9)	28(7) 	-12(8)
F(29)	139(16) 	135(15)	180(20) 	112(17)	-21(14) 	20(11)
F(30)	88(8) 	143(15)	102(11) 	23(9)	-13(7) 	-58(8)
C(64)	77(13) 	92(15)	61(13) 	-5(13)	19(11) 	3(11)
F(65)	139(19) 	320(40)	230(30) 	-50(30)	100(20) 	-120(20)
F(66)	100(16) 	170(20)	250(40) 	100(20)	-90(20) 	-16(14)
C(67)	95(19) 	140(30)	87(18) 	0(17)	28(16) 	-53(19)
F(68)	200(30) 	200(20)	100(19) 	10(15)	85(17) 	-71(18)
F(69)	180(20) 	103(13)	330(40) 	114(17)	130(20) 	65(14)
F(70)	165(17) 	103(11)	137(15) 	7(11)	34(13) 	-75(11)
O(31)	62(3) 	51(3)	69(3) 	22(2)	6(2) 	14(2)
O(32)	58(3) 	46(2)	65(3) 	15(2)	10(2) 	10(2)
C(33)	63(4) 	41(3)	44(3) 	20(3)	7(3) 	2(3)
C(34)	91(6) 	58(4)	58(4) 	22(4)	-5(4) 	9(4)
F(35)	67(3) 	136(5)	91(4) 	40(4)	6(3) 	-11(3)
F(36)	131(4) 	49(2)	73(3) 	17(2)	-6(3) 	6(2)
C(37)	190(13) 	87(7)	65(6) 	38(6)	-16(7) 	3(8)
F(38)	248(9) 	115(5)	112(5) 	78(5)	-4(5) 	33(6)
F(39)	168(6) 	153(6)	64(3) 	47(4)	-28(4) 	-10(5)
F(40)	112(5) 	255(11)	104(5) 	67(6)	49(4) 	15(6)
O(41)	96(3) 	58(3)	69(3) 	38(2)	45(3) 	39(3)
O(42)	86(3) 	58(2)	60(3) 	36(2)	34(2) 	32(2)
C(43)	63(4) 	47(3)	57(4) 	28(3)	24(3) 	17(3)
C(44)	92(5) 	71(4)	72(5) 	44(4)	40(4) 	29(4)
F(45)	233(8) 	68(3)	193(7) 	73(4)	156(6) 	68(4)
F(46)	120(4) 	137(5)	60(3) 	17(3)	24(3) 	56(4)
C(47)	87(6) 	120(8)	102(7) 	60(7)	38(6) 	53(6)
F(48)	140(5) 	151(6)	151(6) 	75(5)	94(5) 	98(5)
F(49)	108(5) 	114(5)	321(13) 	96(7)	119(7) 	33(4)
F(50)	146(7) 	359(16)	97(5) 	48(7)	9(5) 	157(9)
O(51)	52(2) 	56(2)	58(2) 	28(2)	13(2) 	16(2)
O(52)	60(3) 	104(4)	185(7) 	111(5)	33(4) 	28(3)
C(53)	51(3) 	53(3)	69(4) 	37(3)	17(3) 	15(3)
C(54)	62(4) 	89(6)	102(6) 	57(5)	7(4) 	7(4)
F(55)	138(5) 	68(3)	179(7) 	52(4)	-15(5) 	33(3)
F(56)	67(3) 	148(5)	135(5) 	83(4)	39(3) 	51(3)
C(57)	98(8) 	203(15)	122(10) 	116(11)	17(7) 	13(9)
F(58)	213(10) 	137(7)	117(6) 	-7(5)	30(6) 	-24(7)
F(59)	92(4) 	331(13)	128(6) 	137(7)	47(4) 	32(6)
F(60)	158(6) 	220(9)	151(6) 	147(7)	1(5) 	44(6)
______________________________________________________________________________ 
 Table 5.   Hydrogen coordinates ( x 104) and isotropic  displacement parameters (Å2x 10 3)
for pp11a-2000-sch_shape-13b3.
________________________________________________________________________________ 
	x 	y 	z 	U(eq)
________________________________________________________________________________ 
 
________________________________________________________________________________ 
 Table 6.  Torsion angles [°] for pp11a-2000-sch_shape-13b3.
________________________________________________________________ 
Ag(4)-O(11)-C(13)-O(12)	1.7(14)
Ag(4)-O(11)-C(13)-C(14)	-178.9(6)
Ag(1)-O(12)-C(13)-O(11)	-3.2(14)
Ag(1)-O(12)-C(13)-C(14)	177.4(6)
O(11)-C(13)-C(14)-F(16)	-141.6(9)
O(12)-C(13)-C(14)-F(16)	37.9(13)
O(11)-C(13)-C(14)-F(15)	-29.6(12)
O(12)-C(13)-C(14)-F(15)	149.9(8)
O(11)-C(13)-C(14)-C(17)	91.7(12)
O(12)-C(13)-C(14)-C(17)	-88.8(12)
F(16)-C(14)-C(17)-F(19)	174.7(11)
F(15)-C(14)-C(17)-F(19)	64.3(14)
C(13)-C(14)-C(17)-F(19)	-58.5(14)
F(16)-C(14)-C(17)-F(20)	-60.4(14)
F(15)-C(14)-C(17)-F(20)	-170.8(11)
C(13)-C(14)-C(17)-F(20)	66.5(15)
F(16)-C(14)-C(17)-F(18)	52.6(14)
F(15)-C(14)-C(17)-F(18)	-57.8(14)
C(13)-C(14)-C(17)-F(18)	179.5(9)
Ag(2)-O(22)-C(23)-O(21)#2	-22.8(11)
Ag(2)-O(22)-C(23)-C(24)	142.8(9)
Ag(2)-O(22)-C(23)-C(64)	175.8(11)
O(21)#2-C(23)-C(24)-F(25)	7(3)
O(22)-C(23)-C(24)-F(25)	-161(3)
C(64)-C(23)-C(24)-F(25)	111(3)
O(21)#2-C(23)-C(24)-F(26)	123(2)
O(22)-C(23)-C(24)-F(26)	-45(3)
C(64)-C(23)-C(24)-F(26)	-133(3)
O(21)#2-C(23)-C(24)-C(27)	-106.0(15)
O(22)-C(23)-C(24)-C(27)	86.3(17)
C(64)-C(23)-C(24)-C(27)	-1.7(19)
F(25)-C(24)-C(27)-F(30)	50(3)
F(26)-C(24)-C(27)-F(30)	-51(3)
C(23)-C(24)-C(27)-F(30)	174.9(18)
F(25)-C(24)-C(27)-F(28)	180(3)
F(26)-C(24)-C(27)-F(28)	79(2)
C(23)-C(24)-C(27)-F(28)	-55(2)
F(25)-C(24)-C(27)-F(29)	-68(3)
F(26)-C(24)-C(27)-F(29)	-169(2)
C(23)-C(24)-C(27)-F(29)	57(2)
O(21)#2-C(23)-C(64)-F(66)	-3(3)
O(22)-C(23)-C(64)-F(66)	161(2)
C(24)-C(23)-C(64)-F(66)	-93(3)
O(21)#2-C(23)-C(64)-F(65)	-146(4)
O(22)-C(23)-C(64)-F(65)	18(4)
C(24)-C(23)-C(64)-F(65)	124(5)
O(21)#2-C(23)-C(64)-C(67)	105.9(14)
O(22)-C(23)-C(64)-C(67)	-90.7(15)
C(24)-C(23)-C(64)-C(67)	15(2)
F(66)-C(64)-C(67)-F(70)	-60(2)
F(65)-C(64)-C(67)-F(70)	52(3)
C(23)-C(64)-C(67)-F(70)	-178.0(16)
F(66)-C(64)-C(67)-F(69)	-177(2)
F(65)-C(64)-C(67)-F(69)	-65(3)
C(23)-C(64)-C(67)-F(69)	65(2)
F(66)-C(64)-C(67)-F(68)	56(3)
F(65)-C(64)-C(67)-F(68)	169(3)
C(23)-C(64)-C(67)-F(68)	-62(2)
Ag(2)#2-O(31)-C(33)-O(32)	-17.0(9)
Ag(2)#2-O(31)-C(33)-C(34)	164.0(5)
Ag(5)-O(32)-C(33)-O(31)	12.7(9)
Ag(4)-O(32)-C(33)-O(31)	-157.8(5)
Ag(5)-O(32)-C(33)-C(34)	-168.3(5)
Ag(4)-O(32)-C(33)-C(34)	21.2(9)
O(31)-C(33)-C(34)-F(36)	153.4(7)
O(32)-C(33)-C(34)-F(36)	-25.7(10)
O(31)-C(33)-C(34)-C(37)	-78.4(11)
O(32)-C(33)-C(34)-C(37)	102.5(10)
O(31)-C(33)-C(34)-F(35)	36.2(8)
O(32)-C(33)-C(34)-F(35)	-142.9(6)
F(36)-C(34)-C(37)-F(38)	-175.5(10)
F(35)-C(34)-C(37)-F(38)	-62.8(13)
C(33)-C(34)-C(37)-F(38)	56.3(16)
F(36)-C(34)-C(37)-F(39)	-52.7(15)
F(35)-C(34)-C(37)-F(39)	60.0(13)
C(33)-C(34)-C(37)-F(39)	179.1(10)
F(36)-C(34)-C(37)-F(40)	65.0(10)
F(35)-C(34)-C(37)-F(40)	177.7(8)
C(33)-C(34)-C(37)-F(40)	-63.2(11)
Ag(3)-O(42)-C(43)-O(41)#3	1.1(10)
Ag(3)-O(42)-C(43)-C(44)	179.4(4)
O(41)#3-C(43)-C(44)-F(46)	-146.6(7)
O(42)-C(43)-C(44)-F(46)	34.9(9)
O(41)#3-C(43)-C(44)-F(45)	-29.4(10)
O(42)-C(43)-C(44)-F(45)	152.2(7)
O(41)#3-C(43)-C(44)-C(47)	90.8(8)
O(42)-C(43)-C(44)-C(47)	-87.6(8)
F(46)-C(44)-C(47)-F(50)	179.0(10)
F(45)-C(44)-C(47)-F(50)	65.7(12)
C(43)-C(44)-C(47)-F(50)	-56.3(12)
F(46)-C(44)-C(47)-F(49)	-62.8(11)
F(45)-C(44)-C(47)-F(49)	-176.1(9)
C(43)-C(44)-C(47)-F(49)	61.9(11)
F(46)-C(44)-C(47)-F(48)	56.8(10)
F(45)-C(44)-C(47)-F(48)	-56.6(10)
C(43)-C(44)-C(47)-F(48)	-178.6(7)
Ag(5)-O(52)-C(53)-O(51)	-22.7(11)
Ag(6)-O(52)-C(53)-O(51)	150.7(16)
Ag(5)-O(52)-C(53)-C(54)	163.7(6)
Ag(6)-O(52)-C(53)-C(54)	-23(2)
Ag(4)-O(51)-C(53)-O(52)	-50.3(9)
Ag(2)-O(51)-C(53)-O(52)	61.8(10)
Ag(3)-O(51)-C(53)-O(52)	-175.7(6)
Ag(4)-O(51)-C(53)-C(54)	123.2(6)
Ag(2)-O(51)-C(53)-C(54)	-124.7(6)
Ag(3)-O(51)-C(53)-C(54)	-2.2(10)
O(52)-C(53)-C(54)-F(55)	144.3(9)
O(51)-C(53)-C(54)-F(55)	-30.0(11)
O(52)-C(53)-C(54)-F(56)	26.9(11)
O(51)-C(53)-C(54)-F(56)	-147.5(7)
O(52)-C(53)-C(54)-C(57)	-95.8(10)
O(51)-C(53)-C(54)-C(57)	89.9(10)
F(55)-C(54)-C(57)-F(59)	74.5(14)
F(56)-C(54)-C(57)-F(59)	-174.7(12)
C(53)-C(54)-C(57)-F(59)	-49.1(16)
F(55)-C(54)-C(57)-F(60)	-54.4(12)
F(56)-C(54)-C(57)-F(60)	56.4(14)
C(53)-C(54)-C(57)-F(60)	-178.0(9)
F(55)-C(54)-C(57)-F(58)	-173.8(10)
F(56)-C(54)-C(57)-F(58)	-63.0(13)
C(53)-C(54)-C(57)-F(58)	62.6(12)
________________________________________________________________ 
Symmetry transformations used to generate equivalent atoms: 
#1 -x,-y,-z    #2 -x,-y-1,-z-1    #3 -x,-y,-z-1      
#4 -x-1,-y-1,-z-1      

 Table 7.  Hydrogen bonds for pp11a-2000-sch_shape-13b3  [Å and °].
____________________________________________________________________________ 
D-H...A	d(D-H)	d(H...A)	d(D...A)	<(DHA)
____________________________________________________________________________ 
 
 
