# Supplementary material for: Studies on Silver Ions Releasing Processes and Mechanical Properties of Surface-Modified Titanium Alloy Implants
Source: Int J Mol Sci. 2018 Dec 9;19(12):3962. doi: 10.3390/ijms19123962 (PMC6321524; doi:10.3390/ijms19123962)

checkCIF/PLATON report

Structure factors have been supplied for datablock(s) pp11a-2000-sch_shape

THIS REPORT IS FOR GUIDANCE ONLY. IF USED AS PART OF A REVIEW PROCEDURE FOR PUBLICATION, IT SHOULD NOT REPLACE THE EXPERTISE OF AN EXPERIENCED CRYSTALLOGRAPHIC REFEREE.

No syntax errors found.   CIF dictionary   Interpreting this report

Datablock: pp11a-2000-sch_shape

```
Bond precision:  C-C = 0.0144 A          Wavelength=0.71073

Cell:          a=11.3277(5)       b=13.0765(5)      c=13.7547(5)
               alpha=116.746(4)   beta=100.869(3)   gamma=99.819(3)
Temperature:   293 K
```

	Calculated	Reported
Volume	1709.37(15)	1709.36(13)
Space group	P -1	P -1
Hall group	-P 1	-P 1
Moiety formula	C15 Ag5 F25 O13	5(Ag 1+), 3(O H2), 5(C3 F5 O2 1-)
Sum formula	C15 Ag5 F25 O13	C15 H6 Ag5 F25 O13
Mr	1402.50	1408.55
Dx,g cm-3	2.725	2.737
Z	2	2
Mu (mm-1)	3.005	3.005
F000	1308.0	1320.0
F000’	1300.23	
h,k,lmax	14,16,17	14,16,17
Nref	6997	6960
Tmin,Tmax	0.197,0.319	0.279,0.395
Tmin’	0.165	

```
Correction method= # Reported T Limits: Tmin=0.279 Tmax=0.395
AbsCorr = NUMERICAL

Data completeness= 0.995          Theta(max)= 26.371

R(reflections)= 0.0474( 5339)     wR2(reflections)= 0.1439( 6960)

S = 1.043                 Npar= 592
```

The following ALERTS were generated. Each ALERT has the format
test-name_ALERT_alert-type_alert-level.
Click on the hyperlinks for more details of the test.

Alert level B

PLAT230_ALERT_2_B Hirshfeld Test Diff for F40 -- C37 .. 11.4 s.u.

Alert level C

PLAT041_ALERT_1_C Calc. and Reported SumFormula Strings Differ Please Check
PLAT043_ALERT_1_C Calculated and Reported Mol. Weight Differ by .. 6.05 Check
PLAT068_ALERT_1_C Reported F000 Differs from Calcd (or Missing)... Please Check
PLAT215_ALERT_3_C Disordered F25 has ADP max/min Ratio ..... 3.7 Note
PLAT215_ALERT_3_C Disordered F26 has ADP max/min Ratio ..... 3.6 Note
PLAT215_ALERT_3_C Disordered F65 has ADP max/min Ratio ..... 3.7 Note
PLAT215_ALERT_3_C Disordered C27 has ADP max/min Ratio ..... 3.5 Note
PLAT220_ALERT_2_C Non-Solvent Resd 1 C Ueq(max)/Ueq(min) Range 3.2 Ratio
PLAT220_ALERT_2_C Non-Solvent Resd 1 F Ueq(max)/Ueq(min) Range 3.3 Ratio
PLAT234_ALERT_4_C Large Hirshfeld Difference F49 -- C47 .. 0.17 Ang.
PLAT234_ALERT_4_C Large Hirshfeld Difference F58 -- C57 .. 0.25 Ang.
PLAT234_ALERT_4_C Large Hirshfeld Difference F59 -- C57 .. 0.17 Ang.
PLAT234_ALERT_4_C Large Hirshfeld Difference F60 -- C57 .. 0.20 Ang.
PLAT234_ALERT_4_C Large Hirshfeld Difference C53 -- C54 .. 0.16 Ang.
PLAT241_ALERT_2_C High ’MainMol’ Ueq as Compared to Neighbors of Ag1 Check
PLAT241_ALERT_2_C High ’MainMol’ Ueq as Compared to Neighbors of O52 Check
PLAT242_ALERT_2_C Low ’MainMol’ Ueq as Compared to Neighbors of Ag5 Check
PLAT242_ALERT_2_C Low ’MainMol’ Ueq as Compared to Neighbors of O21 Check
PLAT242_ALERT_2_C Low ’MainMol’ Ueq as Compared to Neighbors of C14 Check
PLAT242_ALERT_2_C Low ’MainMol’ Ueq as Compared to Neighbors of C23 Check
PLAT242_ALERT_2_C Low ’MainMol’ Ueq as Compared to Neighbors of C33 Check
PLAT242_ALERT_2_C Low ’MainMol’ Ueq as Compared to Neighbors of C34 Check
PLAT242_ALERT_2_C Low ’MainMol’ Ueq as Compared to Neighbors of C44 Check
PLAT242_ALERT_2_C Low ’MainMol’ Ueq as Compared to Neighbors of C53 Check
PLAT242_ALERT_2_C Low ’MainMol’ Ueq as Compared to Neighbors of C54 Check
PLAT342_ALERT_3_C Low Bond Precision on C-C Bonds .... 0.01438 Ang.
PLAT790_ALERT_4_C Centre of Gravity not Within Unit Cell: Resd. # 1 Note
C15 Ag5 F25 O13
PLAT906_ALERT_3_C Large K value in the Analysis of Variance ...... 3.570 Check
PLAT978_ALERT_2_C Number C-C Bonds with Positive Residual Density. 0 Note

---

Alert level G

FORMU01_ALERT_2_G There is a discrepancy between the atom counts in the
_chemical_formula_sum and the formula from the _atom_site* data.
Atom count from _chemical_formula_sum:C15 H6 Ag5 F25 O13
Atom count from the _atom_site data: C15 Ag5 F25 O13
CELLZ01_ALERT_1_G Difference between formula and atom_site contents detected.
CELLZ01_ALERT_1_G WARNING: H atoms missing from atom site list. Is this intentional?
From the CIF: _cell_formula_units_Z 2
From the CIF: _chemical_formula_sum C15 H6 Ag5 F25 O13
TEST: Compare cell contents of formula and atom_site data

atom	Z*formula	cif sites	diff
C	30.00	30.00	0.00
H	12.00	0.00	12.00
Ag	10.00	10.00	0.00
F	50.00	50.00	0.00
O	26.00	26.00	0.00

PLAT002_ALERT_2_G Number of Distance or Angle Restraints on AtSite 17 Note
PLAT004_ALERT_5_G Polymeric Structure Found with Maximum Dimension 3 Info

```
PLAT040_ALERT_1_G No H-atoms in this Carbon Containing Compound ..     Please Check
PLAT042_ALERT_1_G Calc. and Reported MoietyFormula Strings  Differ     Please Check
PLAT152_ALERT_1_G The Supplied and Calc. Volume s.u. Differ by ...          2 Units
PLAT172_ALERT_4_G The CIF-Embedded .res File Contains DFIX Records          6 Report
PLAT173_ALERT_4_G The CIF-Embedded .res File Contains DANG Records          2 Report
PLAT199_ALERT_1_G Reported _cell_measurement_temperature ..... (K)        293 Check
PLAT200_ALERT_1_G Reported   _diffrn_ambient_temperature ..... (K)        293 Check
PLAT232_ALERT_2_G Hirshfeld Test Diff (M-X)  Ag6    --  O21     ..       13.7 s.u.
PLAT232_ALERT_2_G Hirshfeld Test Diff (M-X)  Ag6    --  O52     ..        9.4 s.u.
PLAT232_ALERT_2_G Hirshfeld Test Diff (M-X)  Ag6    --  O9_a    ..        6.8 s.u.
PLAT242_ALERT_2_G Low    'MainMol' Ueq as Compared to Neighbors of        C17 Check
PLAT242_ALERT_2_G Low    'MainMol' Ueq as Compared to Neighbors of        C37 Check
PLAT242_ALERT_2_G Low    'MainMol' Ueq as Compared to Neighbors of        C47 Check
PLAT242_ALERT_2_G Low    'MainMol' Ueq as Compared to Neighbors of        C57 Check
PLAT300_ALERT_4_G Atom Site Occupancy of Ag6     is Constrained at        0.5 Check
PLAT300_ALERT_4_G Atom Site Occupancy of F25     is Constrained at        0.5 Check
PLAT300_ALERT_4_G Atom Site Occupancy of F26     is Constrained at        0.5 Check
PLAT300_ALERT_4_G Atom Site Occupancy of F28     is Constrained at        0.5 Check
PLAT300_ALERT_4_G Atom Site Occupancy of F29     is Constrained at        0.5 Check
PLAT300_ALERT_4_G Atom Site Occupancy of F30     is Constrained at        0.5 Check
PLAT300_ALERT_4_G Atom Site Occupancy of F65     is Constrained at        0.5 Check
PLAT300_ALERT_4_G Atom Site Occupancy of F66     is Constrained at        0.5 Check
PLAT300_ALERT_4_G Atom Site Occupancy of F68     is Constrained at        0.5 Check
PLAT300_ALERT_4_G Atom Site Occupancy of F69     is Constrained at        0.5 Check
PLAT300_ALERT_4_G Atom Site Occupancy of F70     is Constrained at        0.5 Check
PLAT300_ALERT_4_G Atom Site Occupancy of C24     is Constrained at        0.5 Check
PLAT300_ALERT_4_G Atom Site Occupancy of C27     is Constrained at        0.5 Check
PLAT300_ALERT_4_G Atom Site Occupancy of C64     is Constrained at        0.5 Check
PLAT300_ALERT_4_G Atom Site Occupancy of C67     is Constrained at        0.5 Check
PLAT301_ALERT_3_G Main Residue  Disorder ..............(Resd  1)..         13 % Note
PLAT434_ALERT_2_G Short Inter HL..HL Contact F15    ..  F29     ..       2.80 Ang.
PLAT434_ALERT_2_G Short Inter HL..HL Contact F49    ..  F70     ..       2.73 Ang.
PLAT434_ALERT_2_G Short Inter HL..HL Contact F50    ..  F56     ..       2.84 Ang.
PLAT789_ALERT_4_G Atoms with Negative _atom_site_disorder_group  #          1 Check
PLAT860_ALERT_3_G Number of Least-Squares Restraints .............         20 Note
PLAT898_ALERT_4_G Second Reported H-M Symbol in CIF Ignored ......          ! Check
PLAT910_ALERT_3_G Missing # of FCF Reflection(s) Below Theta(Min)           4 Note
PLAT912_ALERT_4_G Missing # of FCF Reflections Above STh/L=  0.600         33 Note
PLAT933_ALERT_2_G Number of OMIT Records in Embedded .res File ...          1 Note
```

---

   0 **ALERT level A** = Most likely a serious problem - resolve or explain
   1 **ALERT level B** = A potentially serious problem, consider carefully
  29 **ALERT level C** = Check. Ensure it is not caused by an omission or oversight
  44 **ALERT level G** = General information/check it is not something unexpected

  10 ALERT type 1 CIF construction/syntax error, inconsistent or missing data
  28 ALERT type 2 Indicator that the structure model may be wrong or deficient
   9 ALERT type 3 Indicator that the structure quality may be low
  26 ALERT type 4 Improvement, methodology, query or suggestion
   1 ALERT type 5 Informative message, check

It is advisable to attempt to resolve as many as possible of the alerts in all categories. Often the minor alerts point to easily fixed oversights, errors and omissions in your CIF or refinement strategy, so attention to these fine details can be worthwhile. In order to resolve some of the more serious problems it may be necessary to carry out additional measurements or structure refinements. However, the purpose of your study may justify the reported deviations and the more serious of these should normally be commented upon in the discussion or experimental section of a paper or in the "special_details" fields of the CIF. checkCIF was carefully designed to identify outliers and unusual parameters, but every test has its limitations and alerts that are not important in a particular case may appear. Conversely, the absence of alerts does not guarantee there are no aspects of the results needing attention. It is up to the individual to critically assess their own results and, if necessary, seek expert advice.

Publication of your CIF in IUCr journals

A basic structural check has been run on your CIF. These basic checks will be run on all CIFs submitted for publication in IUCr journals (*Acta Crystallographica*, *Journal of Applied Crystallography*, *Journal of Synchrotron Radiation*); however, if you intend to submit to *Acta Crystallographica Section C* or *E* or *IUCrData*, you should make sure that full publication checks are run on the final version of your CIF prior to submission.

Publication of your CIF in other journals

Please refer to the *Notes for Authors* of the relevant journal for any special instructions relating to CIF submission.

PLATON version of 27/03/2017; check.def file version of 24/03/2017

Datablock pp11a-2000-sch_shape - ellipsoid plot

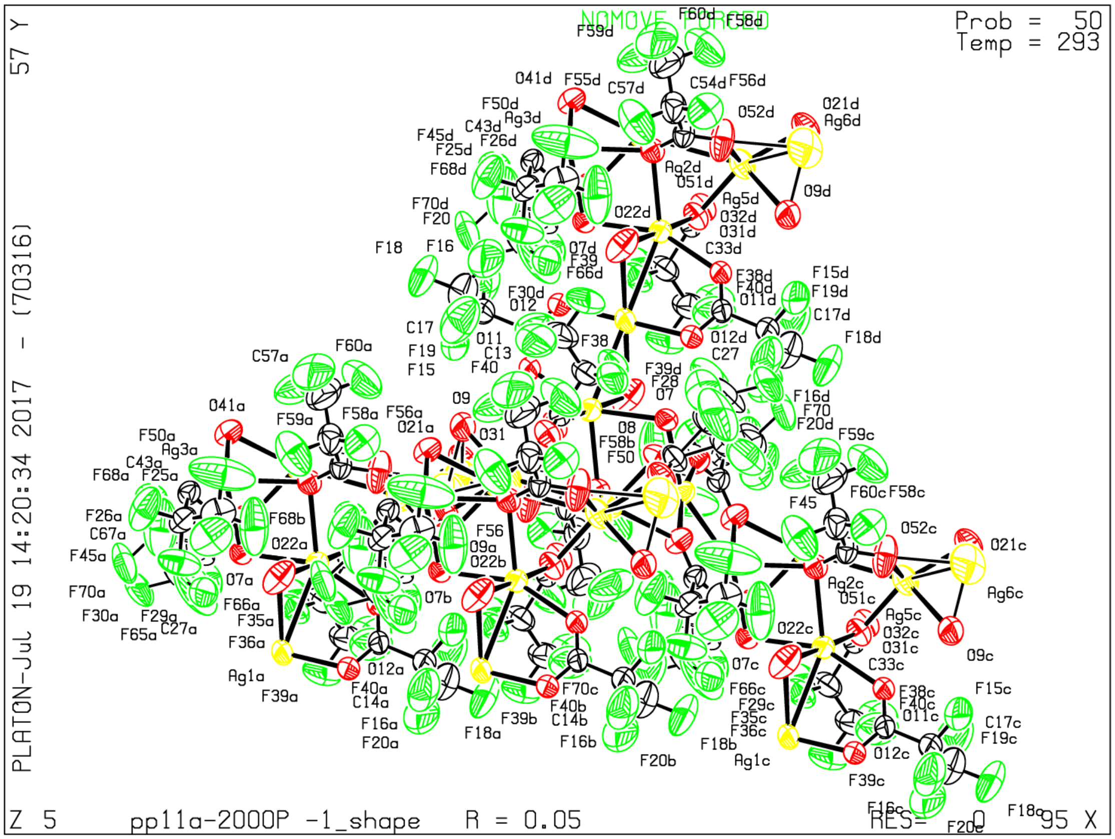

Supplement: Supplementary file 1 [file ijms-19-03962-s001.zip › pp11a-2000-sch_shape-13b3-checkcif.pdf]
